# Filamentous Aggregation of Sequestosome-1/p62 in Brain Neurons and Neuroepithelial Cells upon *Tyr-Cre*-Mediated Deletion of the Autophagy Gene *Atg7*

**DOI:** 10.1007/s12035-018-0996-x

**Published:** 2018-03-17

**Authors:** Supawadee Sukseree, Lajos László, Florian Gruber, Sophie Bergmann, Marie Sophie Narzt, Ionela Mariana Nagelreiter, Romana Höftberger, Kinga Molnár, Günther Rauter, Thomas Birngruber, Lionel Larue, Gabor G. Kovacs, Erwin Tschachler, Leopold Eckhart

**Affiliations:** 1 Research Division of Biology and Pathobiology of the Skin, Department of Dermatology, Medical University of Vienna, Lazarettgasse 14, 1090 Vienna, Austria; 2 Department of Anatomy, Cell and Developmental Biology, Eötvös Loránd University, Budapest, Hungary; 3 Christian Doppler Laboratory on Biotechnology of Skin Aging, Vienna, Austria; 4 Institute of Neurology, Medical University of Vienna, Vienna, Austria; 5 Division of Biomedical Research, Medical University of Graz, Graz, Austria; 6 Joanneum Research, Health - Institute for Biomedicine and Health Sciences, Graz, Austria; 7 Institut Curie, INSERM U1021, CNRS UMR3347, Normal and Pathological Development of Melanocytes, PSL Research University, Orsay, France; 8 INSERM, Orsay, France; 9 Equipe labellisée – Ligue Nationale contre le Cancer, Université Paris 11, Orsay, France

**Keywords:** Autophagy, Protein aggregation, Sequestosome-1, p62, Cortex, Ependyma, Choroid plexus

## Abstract

Defects in autophagy and the resulting deposition of protein aggregates have been implicated in aging and neurodegenerative diseases. While gene targeting in the mouse has facilitated the characterization of these processes in different types of neurons, potential roles of autophagy and accumulation of protein substrates in neuroepithelial cells have remained elusive. Here we report that *Atg7*^*f/f*^ *Tyr-Cre* mice, in which *autophagy-related 7* (*Atg7*) is conditionally deleted under the control of the tyrosinase promoter, are a model for accumulations of the autophagy adapter and substrate sequestosome-1/p62 in both neuronal and neuroepithelial cells. In the brain of *Atg7*^*f/f*^ *Tyr-Cre* but not of fully autophagy competent control mice, p62 aggregates were present in sporadic neurons in the cortex and other brain regions as well in epithelial cells of the choroid plexus and the ependyma. Western blot analysis confirmed a dramatic increase of p62 abundance and formation of high-molecular weight species of p62 in the brain of *Atg7*^*f/f*^ *Tyr-Cre* mice relative to *Atg7*^*f/f*^ controls. Immuno-electron microscopy showed that p62 formed filamentous aggregates in neurons and ependymal cells. p62 aggregates were also highly abundant in the ciliary body in the eye. *Atg7*^*f/f*^ *Tyr-Cre* mice reached an age of more than 2 years although neurological defects manifesting in abnormal hindlimb clasping reflexes were evident in old mice. These results show that p62 filaments form in response to impaired autophagy in vivo and suggest that *Atg7*^*f/f*^ *Tyr-Cre* mice are a model useful to study the long-term effects of autophagy deficiency on the homeostasis of different neuroectoderm-derived cells.

**Electronic supplementary material:**

The online version of this article (10.1007/s12035-018-0996-x) contains supplementary material, which is available to authorized users.

## Introduction

Autophagy is a mechanism for the delivery of cell components to lysosomes for hydrolytic degradation. The main type of autophagy is macroautophagy which involves the formation of double-membraned vesicles, known as autophagosomes, around substrates. A set of autophagy-related genes, such as *Atg5* and *Atg7*, is essential for this process, and deletion of these genes suppresses autophagy in mice [1, 2]. Adaptor proteins such as sequestosome 1, also known as p62 [4], differentially bind to autophagy substrates and introduce specificity into the degradation process [3]. Autophagy removes many types of protein aggregates, dysfunctional organelles, and other potentially dangerous cell components but also contributes to the recycling of macromolecules to ensure cellular homeostasis [3, 5–8].

Sequestosome-1/p62 is a multifunctional protein comprising domains that bind to the mammalian Atg8 homolog microtubule-associated protein 1 light chain 3 (LC3), which mediates docking of autophagy substrates to the forming autophagosome, ubiquitinated proteins, and the Nrf2 regulator Keap1 [4]. Via its N-terminal Phox and Bem1p (PB1) domain, p62 is able to self-oligomerize in the form of filaments [4]. Suppression of autophagy results in the intracellular accumulation of p62 [4]. p62 is present in neurofibrillary tangles in Alzheimer’s disease and Lewy bodies in Parkinson’s disease [9]. Together with reports about the decline of autophagic activity in aged organs and impaired clearance of autophagosomes in neurodegenerative diseases, aberrant processing of p62 in diseased tissues has suggested a particularly important role of autophagy in the aging brain [10–12]. Accordingly, the pharmacological inducer of autophagy, rapamycin, has been suggested as therapeutic agent for aging-associated neurodegeneration [13, 14].

Cell types of different functions and turnover rates vary in their dependence on autophagy for the elimination of damaged organelles and potentially harmful protein aggregates as well as for recycling of building blocks of macromolecules [3, 8, 15]. While constitutive deletion of either *Atg5* or *Atg7* leads to perinatal lethality in mice [16, 17], cell type-specific deletions of autophagy genes via the Cre-loxP system allows to inactivate autophagy in a targeted manner and to determine whether lack of autophagy plays essential roles in these specific cells [1, 2].

In previous studies, we have generated *Atg7*^*f/f*^ *Tyr-Cre* mice for the investigation of the role of autophagy in pigment cells [18–20]. The *Tyr-Cre* gene utilizes promoter and enhancer elements from *tyrosinase* (*Tyr*), a gene encoding the enzyme that converts tyrosine to melanin via tyrosine hydroxylase and dopa oxidase catalytic activities in pigment cells. The *Tyr* promoter drives the expression of a transgene encoding the Cre recombinase, which deletes the region between two loxP sites. The target sites have been introduced into an essential part of the autophagy gene *Atg7* [17], so that the expression of Cre in cells with *Tyr* promoter activity leads to the permanent inactivation of *Atg7*. When these cells proliferate, Atg7-dependent autophagy remains suppressed in all progeny cells. *Atg7*^*f/f*^ *Tyr-Cre* mice show mild hypopigmentation of hair and tail skin [18] but otherwise appear phenotypically normal. Autophagy is also suppressed in the retinal pigment epithelium of *Atg7*^*f/f*^ *Tyr-Cre* mice leading to the accumulation of p62 and an increase in the abundance of a degradation-prone variant of retinal pigment epithelium-specific 65 kDa protein (RPE65) [20].

The characterization of mice carrying the *Tyr-Cre* transgene has shown that Cre expression and Cre-mediated gene deletions do not only occur in pigment cells but also in distinct groups of neurons of the developing brain [21, 22]. Specifically, *Tyr-Cre*-mediated gene deletions were reported in the basal forebrain, hippocampus (dentate gyrus pyramidal cell layers), olfactory bulb, the granule cell layer of the lateral cerebellum cortex, sympathetic cephalic ganglia, leptomeninges of the telencephalon, and cranial nerves (V), (VII), and (IX) [22]. By contrast, the neuroepithelial cells of the adult brain such as the ependyma and the choroid plexus epithelium have not been reported to be affected by *Tyr-Cre*-mediated DNA recombination [21, 22].

Here we investigated *Atg7*^*f/f*^ *Tyr-Cre* mice for p62 accumulations signifying suppression of autophagy in non-pigment cells. We show that p62 accumulates in neuroepithelial cells of the ocular ciliary body, the choroid plexus and the ependyma as well as in neurons of the brain. By immunogold labeling and electron microscopy, the ultrastructure of these p62 aggregates is revealed to consist of filaments both in neurons and neuroepithelial cells. Our data establish *Atg7*^*f/f*^ *Tyr-Cre* mice as a model for the study of aging-associated aberrant p62 depositions in cells of the neuroectodermal lineage.

## Material and Methods

### Mice

The generation and maintenance of *Atg7*^*f/f*^ *Tyr-Cre* mice have been reported previously [18]. Briefly, *Atg7*^*f/f*^ mice (kindly provided by Masaaki Komatsu, Tokyo Metropolitan Institute of Medical Science, Tokyo, Japan) were crossed with mice carrying the *Tyr-Cre* transgene [21]. Tissue samples were prepared from age-matched *Atg7*^*f/f*^ *Tyr-Cre* and *Atg7*^*f/f*^ mice. Only hemizygous males and homozygous females were included to avoid possible effects of X chromosome inactivation on the *Tyr-Cre* transgene in heterozygous females [23].

### Immunohistochemical and Immunofluorescence Analysis

For histological investigations, the eyes were enucleated immediately after sacrificing mice. Likewise, the brain and other tissues were prepared. The tissue samples were fixed in 4% paraformaldehyde over night and then embedded in paraffin. Thin-sections were investigated by immunohistochemistry and immunofluorescence labelling according to published protocols [24] with modifications. The sections were incubated with polyclonal rabbit anti-Sqstm1/p62 (MBL International Corporation, dilution, 1:1000) followed by incubation with goat anti-rabbit immunoglobulin conjugated to horseraddish peroxidase for 30 min. In immunofluorescence double labelings, anti-p62 was used besides mouse monoclonal anti-tyrosine hydroxylase (Millipore, MAB318, clone LNC1, 1: 400) and mouse monoclonal anti-ubiquitin (Millipore, 1:500). The following secondary antibodies were used for immunofluorescence labeling: goat anti-rabbit immunoglobulin coupled to Alexa-Fluor 488 (green) or Alexa-Fluor 546 (red) (Molecular Probes, Leiden, The Netherlands), and goat anti-mouse immunoglobulin coupled to Alexa Fluor 546 (Life Technology, 1:500). Counterstaining of nuclei was done with hematoxylin for immunohistochemistry and Hoechst 33258 (Molecular Probes) for immunofluorescence analysis. Isotype antibodies of unrelated specificities were used instead of the primary antibodies in negative control experiments. The labeled sections were photographed under a fluorescence microscope using the Metamorph software.

### Immunogold Labeling and Electron Microscopy

Whole brains were immersely fixed in immune fixative containing 3.2% paraformaldehyde, 0.2% glutaraldehyde, 1% sucrose, and 3 mM CaCl_2_ in 0.1 M Na-cacodylate buffer for overnight incubation at 4 °C. Pieces of 3 × 3 × 3 mm of the lateral ventricle wall were resected. The small tissue blocks were cryoprotected in 30% sucrose in Na-cacodylate for 24 h. The blocks were frozen in liquid nitrogen and subsequently transferred to anhydrous methanol containing 0.5% uranyl-acetate at − 70 °C. After 6 h, the temperature was raised to − 20 °C and the dehydration was continued for 24 h with gentle agitation. Then specimens were infiltrated with pure LR Gold at − 20 °C for 24 h (three incubations of 8 h each) and then polymerized for 96 h at − 20 °C using a DL-103 12 W ultraviolet lamp.

Ultrathin sections were collected on formvar film-coated nickel grids. For epitope retrieval and quenching, the samples were treated with 0.3% Na-borohydride in Tris-buffered saline containing 50 mM NH_4_Cl and 50 mM glycine for 10 min at room temperature. After antigen retrieval, the samples were incubated with affinity-purified rabbit polyclonal anti-p62 antibody (1:100 dilution, overnight at 4 °C), followed by incubation with goat anti-rabbit immunoglobuin secondary antibody conjugated with 10 nm gold particles (Sigma Aldrich, 1:100, 6 h at room temperature). The immuno-labeled sections were counterstained with uranyl acetate and lead citrate prior to investigation with a JEOL JEM-1011 electron microscope.

### Western Blot Analysis

Brains were lysed in a protein extraction buffer containing 50 mM Tris (pH 7.4), 2% SDS, and complete protease inhibitor cocktail (Roche, Mannheim, Germany) and homogenized by sonication. The insoluble debris was removed by centrifugation, and the protein concentration of the supernatant was measured by the bicinchoninic acid (BCA) method (Pierce, Rockford, IL). Western blot analysis was performed as described previously [20]. Twenty microgram protein was loaded per lane on SDS polyacrylamide gels (ExcelGel SDS, gradient 8–18, Amersham Biosciences) on a horizontal electrophoresis system (Amersham Biosciences) and thereafter blotted onto a nitrocellulose membrane. For the detection of specific antigens, the following first step antibodies were used: rabbit polyclonal anti-p62 (BML-PW9860-0100, Enzo Life Sciences, NY, dilution 1:2000), rabbit anti-Atg7 (Sigma, 1:1000), and mouse anti-GAPDH (HyTest Ltd., Finland, 1:2000). As secondary antibodies, goat anti-rabbit immunoglobulin G (IgG) (Bio-Rad Laboratories, CA) and sheep anti-mouse immunoglobulin G (GE Healthcare, UK) antibodies conjugated to horseradish peroxidase were used at a dilution of 1:10000. The bands were revealed with enhanced chemiluminescence reagent (ThermoFisher Scientific).

### RNA Preparation, Reverse Transcription, and Quantitative PCRs

RNA was prepared from brains using the RNeasy Plus Mini kit (Qiagen, Hilden, Germany) according to the manufacturer’s instructions. RNA was reverse-transcribed using the iScript cDNA synthesis kit (Bio-Rad Laboratories, Hercules, CA) according to the manufacturer’s protocol. Quantitative real-time PCRs with SYBR-Green in the LightCycler system (Roche Applied Science, Mannheim, Germany) were performed according to a published protocol [18, 19]. Transcripts of the following genes were amplified with the indicated primers: *Beta-2 microglobulin* (*B2m*) (Mm_B2m_f, 5′-attcacccccactgagactg-3′ and Mm_B2m_r, 5′-tgctatttctttctgcgtgc-3′), *γ-glutamyl cystine ligase modulatory subunit* (*Gclm*) (Mm_Gclm_f, 5′-tggagcagctgtatcagtgg-3′ and Mm_Gclm_r, 5′-agagcagttctttcgggtca-3′), *NAD(P)H:quinone oxidoreductase 1* (*Nqo1*) (Mm_Nqo1_f, 5′-gaagctgcagacctggtgat-3′ and Mm_Nqo1_r, 5′-ttctggaaaggaccgttgtc-3′), and *Sqstm-1*/p62 (Mm_p62_f, 5′-ccagtgatgaggagctgaca-3′ and Mm_p62_r, 5′-tgggcacacactgcacttat-3′) [18].

### Preparation and Quantification of Lipids

Mouse brain tissue (*n* = 4 per genotype) was homogenized in the ninefold volume of methanol/acetic acid (3%)/butylated hydroxytoluene (BHT, as antioxidant, 0.01%). Samples were purified using the liquid-liquid extraction procedure [25] and were reconstituted in 85% aqueous methanol containing 5 mM ammonium formate and 0.1% formic acid. Analysis was performed at FTC-Forensic Toxicological Laboratory, Vienna. Aliquots (10 μl) were injected onto a core-shell type C 18 column (Kinetex 2.6 μm, 50 mm × 3.0 mm ID; Phenomenex, Torrance, CA) kept at 20 °C and using a 1200 series HPLC system (Agilent, Waldbronn, Germany), coupled to a 4000 QTrap triple quadrupole linear ion trap hybrid mass spectrometer system with a Turbo V electrospray ion source (Applied Biosystems, Foster City, CA, USA). Elution was performed according to a published protocol [25]. Detection was carried out in positive ion mode by selected reaction monitoring (SRM) of 99 MS/MS transitions using product ion (m/z 184), the diagnostic fragment for the phosphocholine residue. Data acquisition and instrument control were performed with Analyst software, version 1.6 (Applied Biosystems). Individual values were normalized to the intrinsic 1,2-di-palmitoyl-3-phosphorylcholine (DPPC) for brain extracts. Non-oxidized native lipid species (1-palmitoyl-2-arachidonoyl-sn-glycero-3-phosphorylcholine (PAPC) *m/z* 782; 1-palmitoyl-2-linoleoyl-sn-glycero-3-phosphorylcholine (PLPC) *m/z* 758; 1-stearoyl-2-arachidonoyl-sn-glycero-3-phosphorylcholine (SAPC) *m/z* 810; 1-stearoyl-2-linoleoyl-sn-glycero-3-phosphorylcholine (SLPC) *m/z* 786) and chain fragmented oxidized species (1-palmitoyl-2-(5-oxovaleroyl)-sn-glycero-3-phosphorylcholine (POVPC) *m/z* 594; 1-palmitoyl-2-azelaoyl-sn-glycero-3-phosphorylcholine (PAzPC) *m/z* 666; 1-stearoyl-2-azelaoyl-sn-glycero-3-phosphorylcholine (SAzPC) *m/z* 694 1-palmitoyl-2-glutaroyl-sn-glycero-3-phosphorylcholine (PGPC) *m/z* 610; 1-palmitoyl-2-(oxo-nonanoyl)-sn-glycero-3-phosphorylcholine (PONPC) *m/z* 650 and 1-stearoyl-2-(oxo-nonanoyl)-sn-glycero-3-phosphorylcholine (SONPC) *m/z* 678) were identified as isobaric and co-eluting with commercial standards [25].

### Preparation of CSF and Analysis of Proteins by Electrophoresis

The CSF was prepared according to a published protocol [26]. Proteins were separated by polyacrylamide gel electrophoresis and subjected to silver staining [27].

### Statistics

The statistical significance of differences between sample groups was examined using the two-tailed unpaired Student’s *t* test. *P* values below 0.05 were considered significant.

### Ethics Statement

Mice were maintained and sacrificed by cervical dislocation according to the animal welfare guidelines of the Medical University of Vienna, Austria, as approved by the Ethics Review Committee for Animal Experimentation of the Medical University of Vienna, Austria, and the Federal Ministry of Science, Research and Economy, Austria (GZ 66.009/0255-II/3b/2013). CSF was prepared under approval of the Federal Ministry of Science, Research and Economy, Austria (BMWFW-66.010/0045-WF/V/3b/2015). All methods were performed in accordance with the relevant guidelines and regulations.

## Results

### *Tyr-Cre*-Mediated Deletion of *Atg7* Leads to Accumulation of p62 in Neurons and Neuroepithelial Cells of the Brain

As the *Tyr-Cre* transgene has been reported to be active not only in pigment cells but also in other cells of the neuroectodermal lineage [21, 22], we analyzed the brain of *Atg7*^*f/f*^ *Tyr-Cre* mice and, as control, the brain of fully autophagy-competent *Atg7*^*f/f*^ mice. Both male and female *Atg7*^*f/f*^ *Tyr-Cre* mice were successfully used in breeding, had normal weight, and could be kept up to an age of 2 years. However, *Atg7*^*f/f*^ *Tyr-Cre* mice older than 1.5 years showed abnormal limb-clasping reflexes that were also reported in mouse models of neurodegenerative diseases [1, 2, 28] (Suppl. Fig. S1). To determine possible changes in the central nervous system of *Atg7*^*f/f*^ *Tyr-Cre* mice, brains of young (age 1–2 months) and old (age 14–27 months) mice of both genotypes (*Atg7*^*f/f*^ *Tyr-Cre* and *Atg7*^*f/f*^ mice) were investigated.

**Fig. 1:**
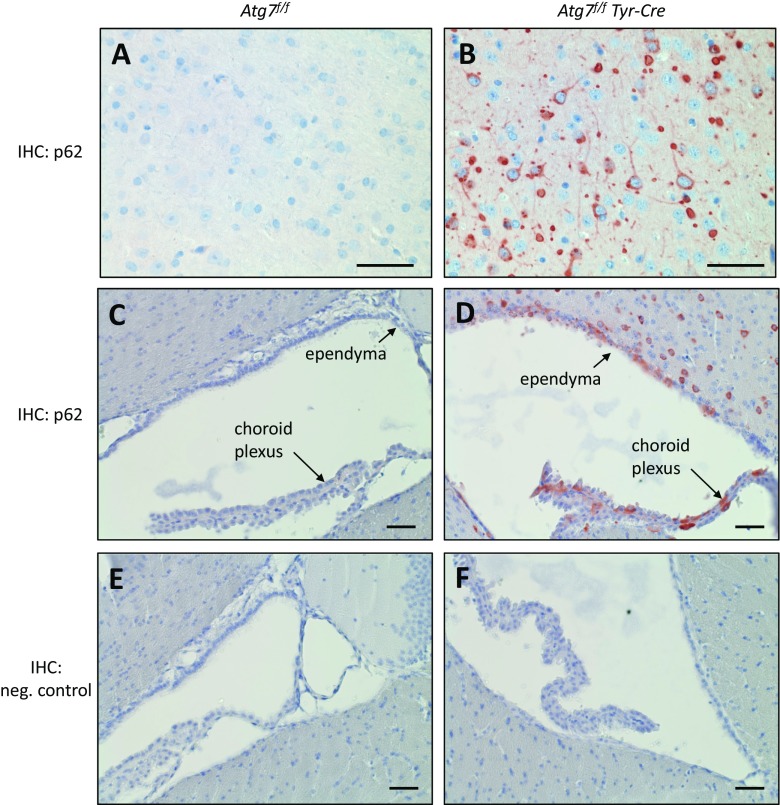
*Tyr-Cre-mediated deletion of Atg7 leads to differential accumulation of p62 in cells of the brain.* p62 was immunohistochemically (IHC) stained (red) in the brain of *Atg7*^*f/f*^ (A, C) and *Atg7*^*f/f*^ *Tyr-Cre* (B, D) mice aged at least 14 months. In negative control experiments the anti-p62 antibody was replaced by equally concentrated antiserum of unrelated specificity (E, F). The images show the cortex (A, C) and the brain regions around the third ventricle (C–F). Scale bars, 50 μm

Immunohistochemistry showed that p62 was present only at minimal amounts in the brain of *Atg7*^*f/f*^ mice whereas it accumulated in various areas of the brain of *Atg7*^*f/f*^ *Tyr-Cre* mice (Fig. 1). The abundance of p62 was massively increased in 2-year-old *Atg7*^*f/f*^ *Tyr-Cre* mice (Fig. 1) but accumulations of p62 were already present in 1-month-old mice of this genotype (Suppl. Fig. S2). Neurons in the cortex (mainly in the frontal and parietal lobe, large spherical inclusions in the pyramidal layer), the basal ganglia, parts of the thalamus, and few neurons in the brain stem contained p62 in the form of aggregates with diameters of up to 12 μm (Fig. 1). Besides p62 accumulations in cell bodies of these regions, round to oval p62 deposits were also present in neuronal processes. By contrast, the hippocampus, leptomeninges, dentate nucleus, and the cerebellum of *Atg7*^*f/f*^ *Tyr-Cre* mice were immunonegative for p62. In the substantia nigra, tyrosine hydroxylase-positive cells which produce neuropigment did not contain accumulations of p62 (Suppl. Fig. S3). Although there are, to the best of our knowledge, no reports about *Tyr-Cre*-mediated recombination in neuroepithelial cells of the brain [21, 22], we detected accumulations of p62 in the ependyma and the choroid plexus epithelium of *Atg7*^*f/f*^ *Tyr-Cre* mice (Fig. 1).

**Fig. 2:**
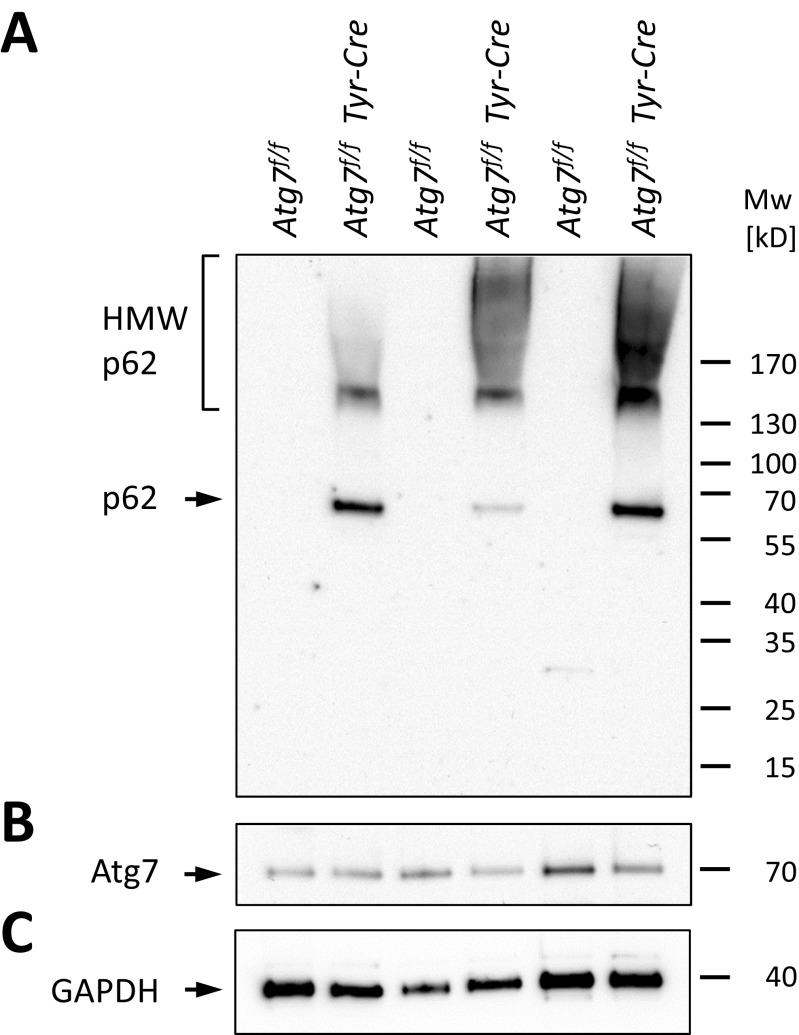
*Western blot analysis shows increase of p62 abundance and formation of high-molecular weight species of p62 in the brain of Atg7*^*f/f*^ *Tyr-Cre mice.* Protein lysates from whole brains of *Atg7*^*f/f*^ and *Atg7*^*f/f*^ *Tyr-Cre* mice (*n* = 3 per genotype, age: 23–26 months) were analyzed by Western blot for p62 (A), Atg7 (B), and GAPDH (C). Positions of molecular weight markers are indicated on the right. HMW, high molecular weight; kD, kilo-Dalton; Mw, molecular weight

Western blot analysis confirmed the strong increase in p62 abundance in the brains of *Atg7*^*f/f*^ *Tyr-Cre* mice relative to that of *Atg7*^*f/f*^ mice (Fig. 2A). While the level of p62 was below the Western blot detection limit in brains of *Atg7*^*f/f*^ mice, p62 was consistently detected at the expected size and in the form of high molecular weight protein species, indicating oligomerization, in the brain of *Atg7*^*f/f*^ *Tyr-Cre* mice. Western blot analysis did not show a difference of total Atg7 amounts in the brain lysates of the two mouse genotypes (Fig. 2B), which was consistent with the finding that the vast majority of cells were not altered with regard to Atg7-dependent p62 degradation in the brain of *Atg7*^*f/f*^ *Tyr-Cre* mice (Fig. 1). In contrast to the elevation of p62 at the protein level, Sqstm1/p62 mRNA was not increased in the brain of *Atg7*^*f/f*^ *Tyr-Cre* mice (Suppl. Fig. S4), suggesting that the accumulation of p62 protein was not driven by enhanced biosynthesis but by reduced degradation. Targets of the autophagy-sensitive Nrf2-mediated stress response such as *Nqo1* and *Gclm1* were not upregulated in the brain of *Atg7*^*f/f*^ *Tyr-Cre* mice (Suppl. Fig. S4).

### *Atg7*^*f/f*^*Tyr-Cre*-Induced Aggregates of p62 Are Not Strictly Associated with Ubiquitin

**Fig. 3:**
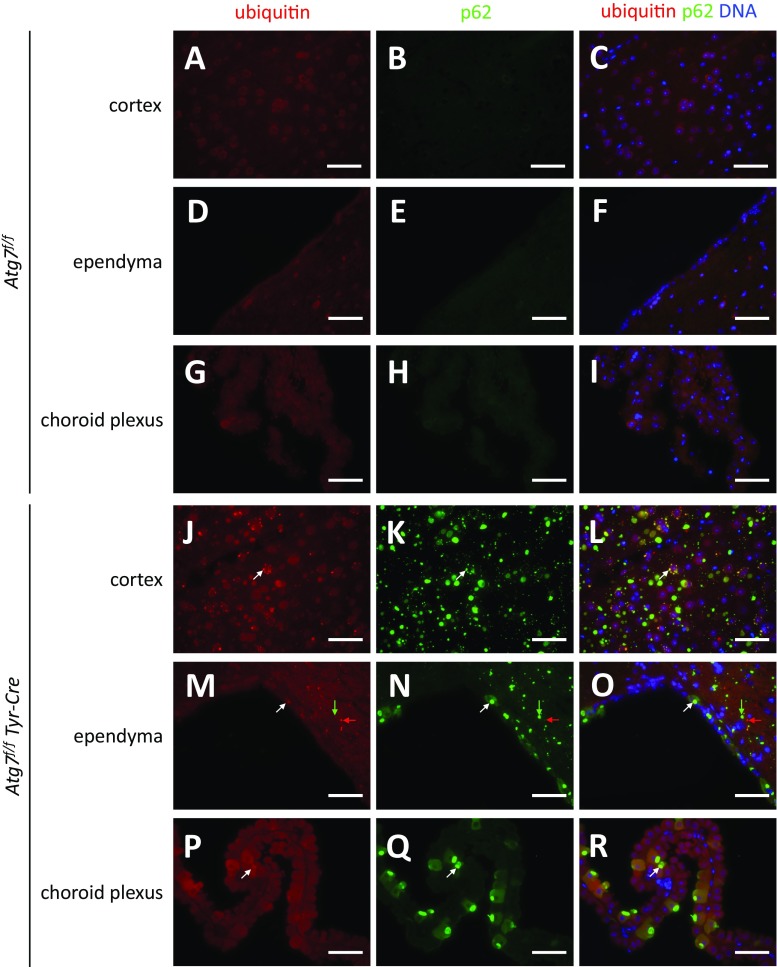
*The accumulation of p62 is only partially linked to accumulation of ubiquitin in Atg7*^*f/f*^ *Tyr-Cre mice.* Brains of *Atg7*^*f/f*^ (A–I) and *Atg7*^*f/f*^ *Tyr-Cre* (J–R) mice (age: 2 years) were sectioned and subjected to double immunolabeling for ubiquitin (red) and p62 (green). Nuclear DNA was labeled with Hoechst dye (blue). White arrows indicate examplary aggregates that were positive for p62 and, weakly, ubiquitin; red arrows indicate an examplary aggregate that was positive only for ubiquitin; and green arrows indicate an examplary aggregate that was positive only for p62. Scale bars, 50 μm

Immunofluorescence analysis confirmed that p62 aggregates were present both in cell bodies and dendrites of many neurons in *Atg7*^*f/f*^ *Tyr-Cre* mice (Fig. 3). Besides aggregates of diameters in the range of 0.5–2 μm, large aggregates of up to 10 μm in diameter were detected in 2-year-old mice (Fig. 3K). A fraction of ependymal and choroid plexus epithelial cells contained elevated amounts of p62 and p62 bodies in *Atg7*^*f/f*^ *Tyr-Cre* but not in *Atg7*^*f/f*^ mice (Fig. 3N, Q). The p62 aggregates in neuroepithelial cells reached sizes similar to those in brain neurons (up to 8 μm in the ependyma and up to 12 μm in the choroid plexus). Interestingly, most of the p62 aggregates were present in the apical cytoplasm of epithelial cells of the choroid plexus (Fig. 3Q) whereas p62 bodies appeared to be randomly distributed in ependymal cells of *Atg7*^*f/f*^ *Tyr-Cre* mice (Fig. 3N). Notably, only a subset of epithelial cells in the ependyma and choroid plexus contained p62 aggregates whereas the others were immunonegative for p62.

Immuno-labeling for ubiquitin showed that some but not all p62 bodies in the brains of *Atg7*^*f/f*^ *Tyr-Cre* mice were associated with immunoreactivity for ubiquitin (Fig. 3). Only in few neurons, mainly located in the cortex (Fig. 3J–L), the relative increase of ubiquitin was as pronounced as that observed for p62, whereas many cells with p62 accumulations did not have increased levels of ubiquitin.

Electrophoretic analysis suggested that the cerebrospinal fluid (CSF), which is secreted by the choroid plexus epithelium and, to a smaller extent, by the ependyma [29], contained the same major protein species at the same relative abundance in *Atg7*^*f/f*^ *Tyr-Cre* and *Atg7*^*f/f*^ mice (Suppl. Fig. S5). Thus, *Tyr-Cre*-mediated deletion of Atg7 caused aberrant accumulation of the autophagy substrate p62 without deleterious effects on the secretory function of neuroepithelial cells.

### Immunogold Electron Microscopy Shows Filamentous Structure of p62 Aggregates in Epithelial Cells of the Ependyma and Neurons of *Atg7*^*f/f*^*Tyr-Cre* Mice

**Fig. 4:**
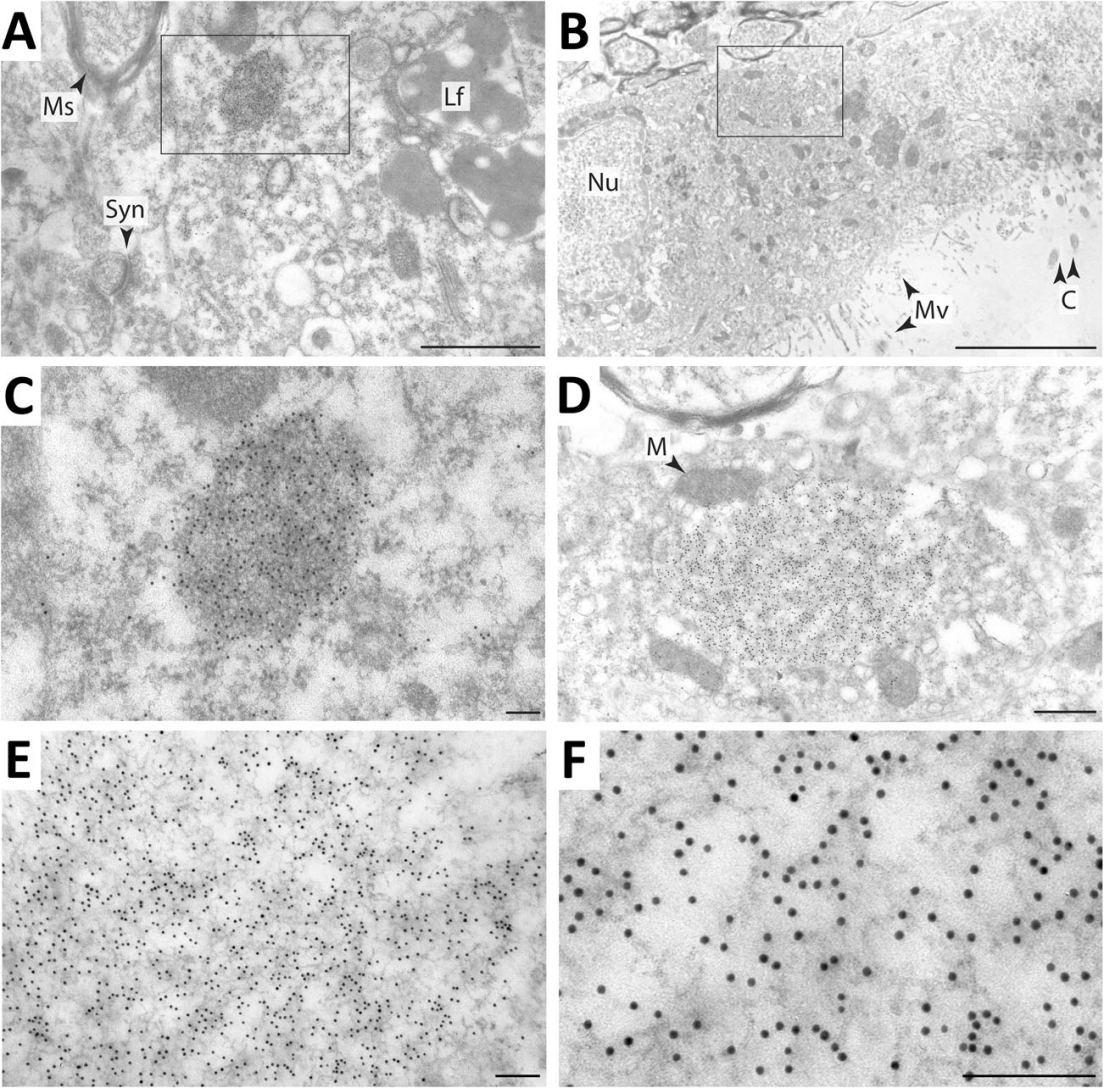
*Immunogold electron microscopy shows filamentous structure of p62 aggregates in neurons and epithelial cells of the ependyma of Atg7*^*f/f*^ *Tyr-Cre mice.* Ultrathin sections of mouse brain were labeled with anti-p62 antibody conjugated to 10-nm gold particles. Electron micrographs of only *Atg7*^*f/f*^ *Tyr-Cre* brains (age: 23 months) are shown whereas *Atg7*^*f/f*^ brain (age: 26 months) showed only sparse immunogold labeling. (A) Electron micrograph of the pericaryonal region of a thalamic neuron containing a p62 body (framed area). (B) Electron micrograph of the ependyma containing a p62 body (framed area). The framed regions of panels A and B are shown at higher magnification in panels C and D. (E, F) The fine filamentous meshwork of a p62 body is shown at high magnifications. Lf, lipofuscin granules; M, mitochondrion; Ms, myelin sheath; Mv, microvilli; Nu, nucleus; Syn, synapse. Scale bars: 1 μm (A); 5 μm (B), 100 nm (C, E, F), 500 nm (D)

Next, we investigated the ultrastructural organization of the p62 accumulations in neurons and neuroepithelial cells of *Atg7*^*f/f*^ *Tyr-Cre* brain. Immunogold electron microscopy showed that p62 was concentrated in aggregates in the cytoplasm of neurons (Fig. 4A, C) and ependymal cell at the ventricle wall (Fig. 4B, D) of *Atg7*^*f/f*^ *Tyr-Cre* mice whereas no or only sparse p62 labels were detected in the brain of *Atg7*^*f/f*^ mice (up to an age of 26 months). The aggregates in neurons and ependymal of *Atg7*^*f/f*^ *Tyr-Cre* mice were composed of electron-dark filaments that were densely decorated with anti-p62 immunogold labels (Fig. 4C–F). The aggregates were not surrounded by a membrane. The organization of the p62 bodies was similar in all affected brain cell types investigated and in mice aged 9 months (Suppl. Figs. S6, S7) and 23 months (Fig. 4).

### *Tyr-Cre*-Mediated Deletion of *Atg7* Is Associated with an Increase in Dicarboxylic Acid-Containing Phospholipids in the Brain

**Fig. 5:**
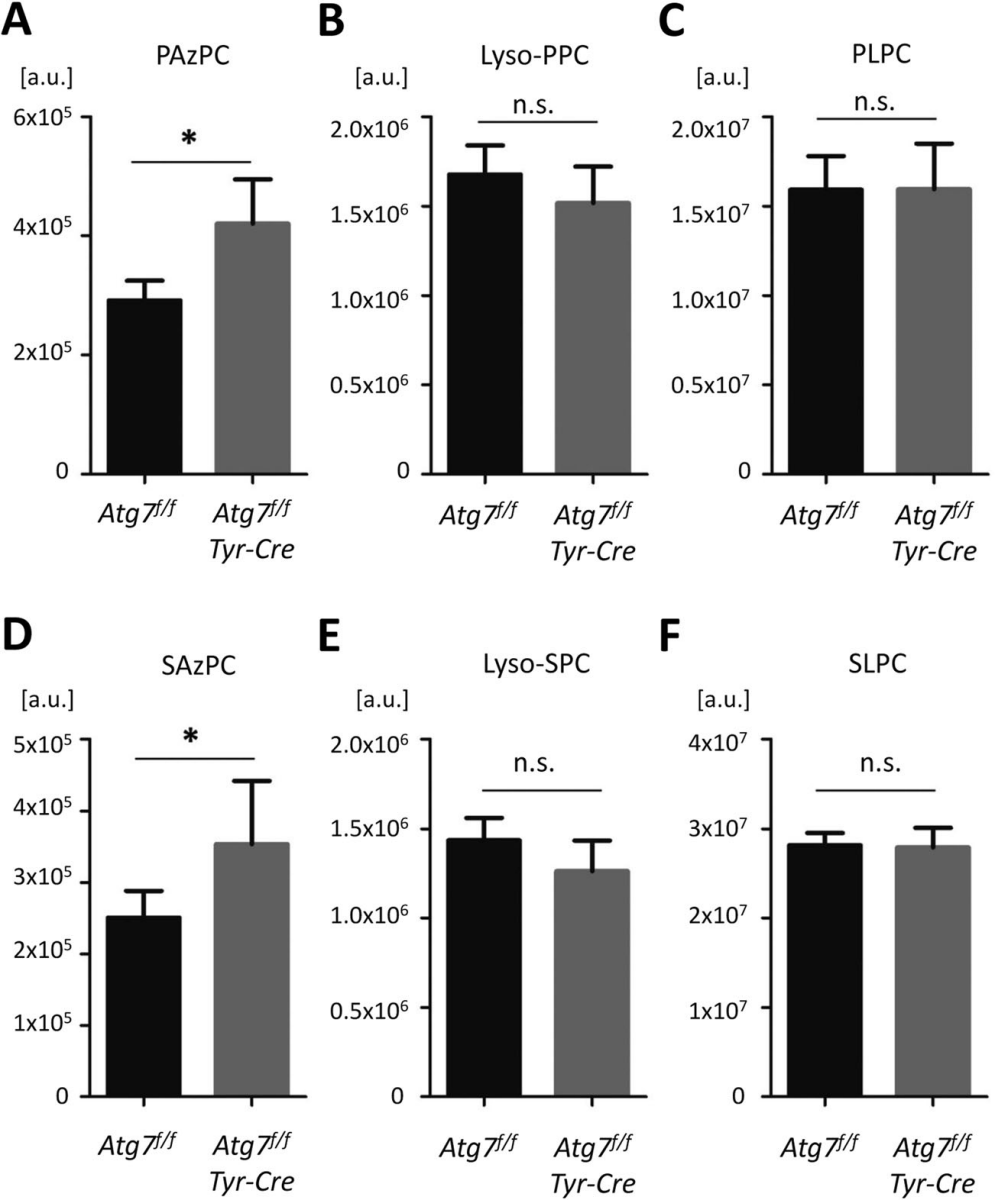
*Tyr-Cre-mediated deletion of Atg7 leads to alterations of the lipid composition in the brain.* Lipids were extracted from the whole brain of *Atg7*^*f/f*^ and *Atg7*^*f/f*^ *Tyr-Cre* mice (age: 1 year) and analyzed as described in the Materials and Methods section. The abundance of individual lipids (A–F) was normalized to DPPC. The bars indicate quantities in arbitrary units and error bars indicate standard deviations. *n* = 4 per genotype. *, significant with *P* < 0.05 (two-sided *t* test). a.u., arbitrary units; Lyso-PPC, 1-palmitoyl-2-hydroxy-*sn*-glycero-3-phosphocholine; Lyso-SPC, 1-stearoyl-2-hydroxy-*sn*-glycero-3-phosphocholine; PAzPC, 1-palmitoyl-2-azelaoyl-*sn*-glycero-3-phosphorylcholine; PLPC, 1-palmitoyl-2-linoleoyl-*sn*-glycero-3-phosphorylcholine; SAzPC, 1-stearoyl-2-azelaoyl-*sn*-glycero-3-phosphorylcholine; SLPC, 1-stearoyl-2-linoleoyl-*sn*-glycero-3-phosphorylcholine

Autophagy contributes to the lipid metabolism of cells [30, 31], and we have recently identified oxidized phospholipid species that accumulated in autophagy-deficient melanocytes that acquired a premature senescent phenotype [19]. We thus applied a recently developed HPLC-MS/MS method to investigate the abundance of selected oxidized phospholipid species in brains from *Atg7*^*f/f*^ and *Atg7*^*f/f*^ *Tyr-Cre* mice. The azelaic acid-containing oxidation products of two major unsaturated phospholipds, PLPC and SLPC, 1-palmitoyl-2-azelaoyl-sn-glycero-3-phosphocholine (PAzPC), and 1-stearoyl-2-azelaoyl-sn-glycero-3-phosphocholine (SAzPC) were significantly increased relative to the saturated internal control lipid, 1-,2-dipalmitoyl-3-phosphocholine (DPPC) (Fig. 5), which was similar to the increase of PAzPC in cultured autophagy-deficient melanocytes of *Atg7*^*f/f*^ *Tyr-Cre* mice [19]. By contrast, the levels of the unoxidized PLPC and SLPC as well as those of Lyso-PPC and Lyso-SPC were not significantly different between *Atg7*^*f/f*^ and *Atg7*^*f/f*^ *Tyr-Cre* mice (Fig. 5). Of note, phospholipid-esterified azelaic acid can bind to lysine residues [32] and thereby may contribute to lipoxidative damage and aggregation of proteins in cells affected by *Tyr-Cre*-induced suppression of autophagy.

### p62 Accumulates in the Ciliary Body of the Eye of *Atg7*^*f/f*^*Tyr-Cre* Mice

**Fig. 6:**
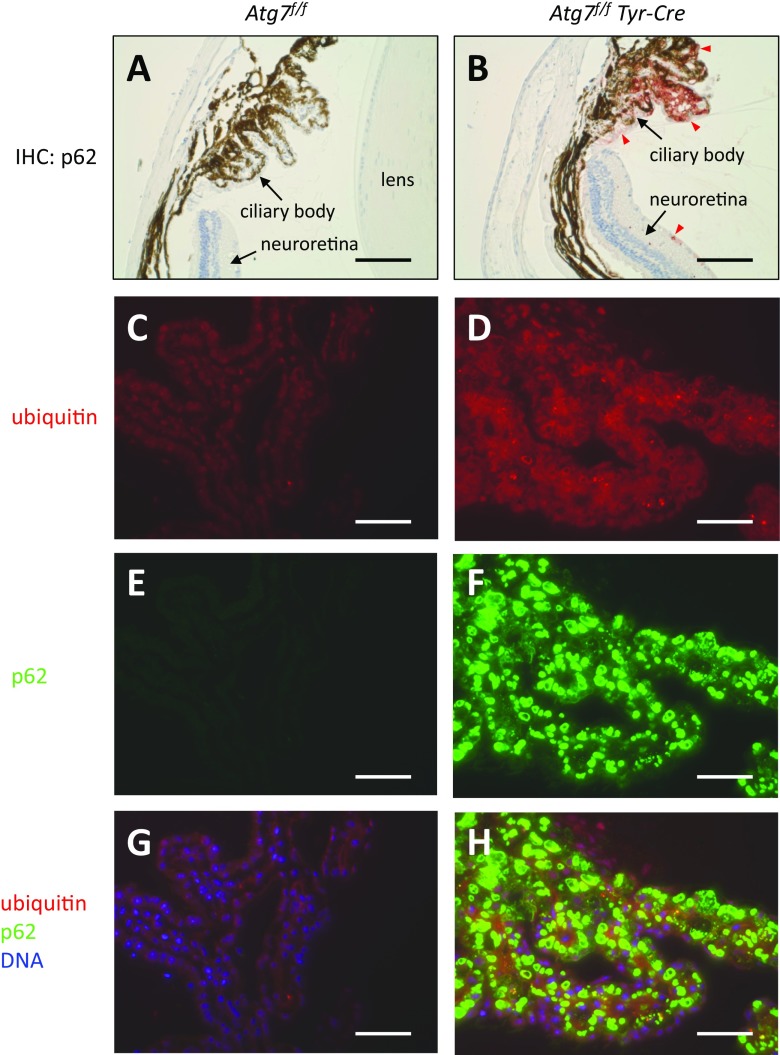
*Tyr-Cre-mediated deletion of Atg7 leads to accumulation of p62 in the ciliary body of the eye.* Eyes of *Atg7*^*f/f*^ (A, C, E, G) and *Atg7*^*f/f*^ *Tyr-Cre* (B, D, F, H) mice were sectioned and subjected to immunohistochemistry (IHC) for p62 (red) (A, B) and to double immunolabeling for ubiquitin (red) and p62 (green) (C–H). Nuclear DNA was labeled with Hoechst dye (blue). Red arrowheads (A, B) indicate p62-positive ciliary body epithelial cells and sparse p62-positive cells in the neuroretina of *Atg7*^*f/f*^ *Tyr-Cre* mice. The images are representative for at least n = 3 per genotype and age group (10 months in panels A and B**,** and 21 months in C**–**H). Scale bars, 100 μm (A, B), 50 μm (C–H)

Outside of the brain, p62 accumulations were detected in skin melanocytes [18], choroid melanocytes of the eye, and retinal pigment epithelial cells [20]. However, the strongest accumulation of p62, as judged from immunohistochemical and immunofluorescence analysis, was found in the epithelial cells of the ciliary body of *Atg7*^*f/f*^ *Tyr-Cre* mice (Fig. 6). p62 formed large inclusions in both pigmented and non-pigmented ciliary body epithelial cells of *Atg7*^*f/f*^ *Tyr-Cre* mice. In approximately half of the *Atg7*^*f/f*^ *Tyr-Cre* mice investigated, p62 accumulations were also present at low abundance in the neuroretina (Fig. 6). Eyes of *Atg7*^*f/f*^ mice did not contain appreciable amounts of p62 (Fig. 6).

**Fig. 7:**
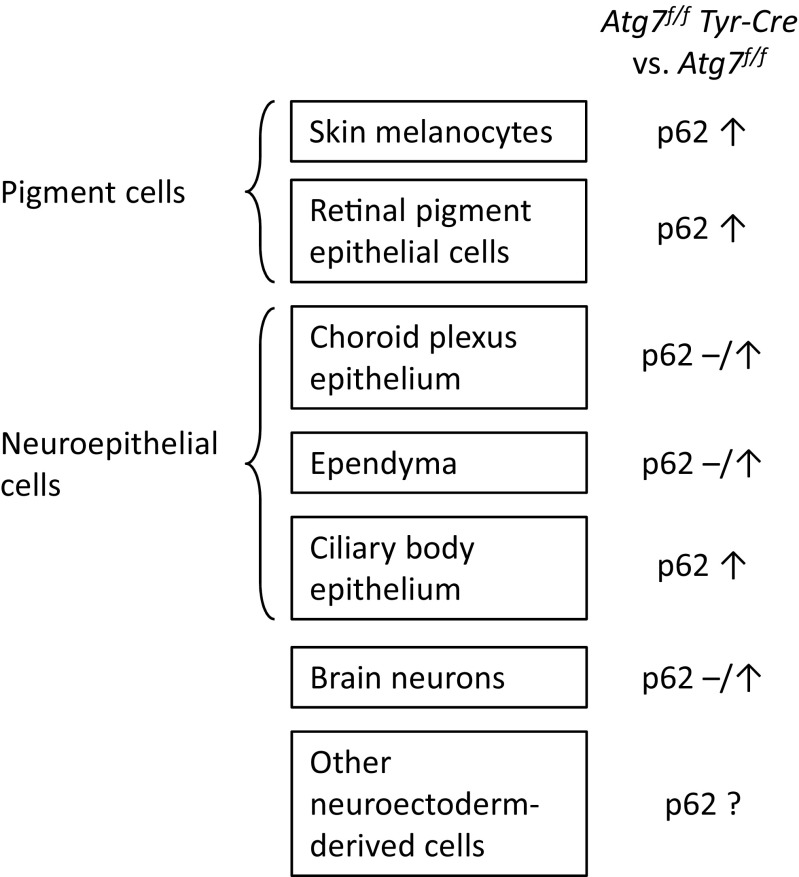
*Summary of differential effects of Tyr-Cre-mediated deletion of Atg7 on p62 abundance in neuroectoderm-derived cell types.* Neuroectoderm-derived cells of *Atg7*^*f/f*^ *Tyr-Cre* and *Atg7*^*f/f*^ (fully autophagy-competent) mice were characterized with regard to the abundance and distribution of p62. The results of previous studies on melanocytes and retinal pigment epithelial cells [18, 20] and of the present study are schematically summarized. ↑, accumulation; −, absent; −/↑, absent and accumulated in different subsets of this cell type

In summary, both pigment cells expressing tyrosinase, such as melanocytes, and other neuroectodermal cells developed accumulations of p62 in response to *Tyr-Cre-*mediated deletion of *Atg7*. This pattern suggested that the activation of the tyrosinase promoter in the *Tyr-Cre* transgene and the subsequent inactivation of *Atg7* occurred in developmental precursor cells which inherit the lack of Atg7-dependent autophagy to their neuronal and neuroepithelial progeny (Fig. 7).

## Discussion

The results of this study establish *Atg7*^*f/f*^ *Tyr-Cre* mice as a model for the study of autophagy-deficiency in non-pigment cells of the neuroectodermal lineage and support the hypothesis of filament formation of endogenous p62 as a mechanism of sequestering p62 and possibly other proteins in autophagy-deficient cells. Given the emerging role of p62 as a multi-functional adapter protein [4, 33], our findings are likely relevant for several aspects of neurobiology.

In the present study, the *Tyr-Cre* transgene [21, 23] was used to delete the floxed alleles of *Atg7*. The transgenic tyrosinase promoter was shown to be active in some neural crest cell precursors of melanocytes, some smooth muscle cells of the heart and cells of the enteric nervous system, but also in the brain [21, 23, 34, 35]. The neuronal expression of *Tyr-Cre* was used to delete *phosphatase and tensin homolog* (*Pten*) in a subset of vagal neural crest cells, resulting in lethal intestinal pseudoobstruction of *Tyr-Cre/° Pten*^*f/f*^ mice [36]. We have previously investigated the effects of *Tyr-Cre* mediated deletion of *Atg7* on skin melanocytes and retinal pigment epithelial cells [18, 20]. *Atg7*^*f/f*^ *Tyr-Cre* mice displayed mild defects in hair pigmentation and alteration in the turnover of the C57BL/6 background-specific M450 variant of RPE65 and reached an age beyond 2 years. Of note, tyrosinase is not involved in the synthesis of neuromelanin, the pigment within the substantia nigra, a region of the midbrain [37], and accordingly, a specific deletion of *Atg7* was not expected in the substantia nigra of *Atg7*^*f/f*^ *Tyr-Cre* mice. However, in line with the reported expression of the *Tyr-Cre* transgene in multiple non-pigmented neuroectodermal cells, we found a diverse set of phenotypically abnormal cells. The present characterization of p62 aggregations in a series of neuroectodermal lineage cells of *Atg7*^*f/f*^ *Tyr-Cre* mice is an important extension of previous studies because it suggests that cell-autonomous effects, such as those on melanocytes, may be accompanied by effects of altered signaling from neurons in *Atg7*^*f/f*^ *Tyr-Cre* mice.

The accumulation of p62 in neurons and neuroepithelial cells of the brain and the eyes of *Atg7*^*f/f*^ *Tyr-Cre* mice indicates that Atg7-dependent autophagy is abrogated in these cells. The reduced degradation of p62 is an accepted in vivo marker of impaired autophagy [38, 39], especially when supported by evidence for lack of transcriptional upregulation of *Sqstm1/p62* expression (Suppl. Fig. S4). Importantly, the subcellular abnormalities in the brain of *Atg7*^*f/f*^ *Tyr-Cre* mice are associated with abnormal hindlimb clasping reflexes at an age of 1.5 years and more in *Atg7*^*f/f*^ *Tyr-Cre* mice (Suppl. Fig. S1) but do not impair the survival of mice and therefore differ from the effects of *Atg7* and *Atg5* deletions in all neurons which are lethal within 4–28 weeks after birth of mice [1, 2]. Thus, *Atg7*^*f/f*^ *Tyr-Cre* mice extend the list of viable mouse lines carrying deletions of *Atg7* in distinct sets of brain neurons [40–49]. Accordingly, *Atg7*^*f/f*^ *Tyr-Cre* mice will be available for studying the impact of lack of autophagy and aberrant accumulation of p62 in neurons of the brain in future studies.

Unexpectedly, neuroepithelial cells of the choroid plexus, the ependyma, and the ocular ciliary body were also affected by *Tyr-Cre*-driven inactivation of *Atg7*. To the best of our knowledge, conditional suppression of autophagy has not been reported previously in these cell types. The structure of p62 bodies in ependymal cells was similar to that in neurons, and the p62 aggregates in the ciliary body were even larger than those in neurons. The neuroepithelia of the choroid plexus and the ciliary body do not only share common embryological origins [50]; they also have similar functions. Both of these epithelia secrete liquids, namely the cerebrospinal fluid and the aqueous humor of the eye, respectively. As the secretion rates of these epithelia control the intracranial pressure and the intraocular pressure, defects of the choroid plexus epithelium and the ciliary body epithelium may be medically relevant. It remains to be investigated whether *Tyr-Cre*-driven gene recombination can be used to study functional parameters pertaining to the etiology of hydrocephalus or glaucoma. Choroid plexus epithelial cells of *Atg7*^*f/f*^ *Tyr-Cre* mice developed p62 bodies predominantly in the cell periphery, reminiscent of the so-called Biondi bodies or Biondi ring tangles that appear during human aging and increase significantly in patients with Alzheimer’s disease [51, 52]. Although the structures of the p62 aggregates in the mutant mice and those of the tangles in humans are different [53], possible similarities in mechanisms of formation should be investigated.

Our data suggest that p62 forms filamentous aggregates when sufficiently high-intracellular concentrations are reached due to lack of autophagic turnover. In *Atg7*^*f/f*^ *Tyr-Cre* mice, p62 was diffusely distributed in melanocytes [18] and some brain neurons (Fig. 1) whereas in most of the affected neurons and neuroepithelial cells, p62 was concentrated in aggregates, also referred to as p62 bodies. The formation of p62 bodies has also been reported for other mouse models of autophagy deficiency and therefore appears to be a characteristic feature of p62 [54]. Our investigation of the ultrastructural organization of p62 bodies demonstrates filaments of uniform thickness that were densely bound by anti-p62 antibodies. Previous studies have suggested that protein aggregates containing endogenous p62 have a filamentous organization, e.g., in dendrites of dopaminergic neurons of mice that carry a tyrosine hydroxylase (TH) cell-specific deletion of *Atg7* (*Atg7*^*f/f*^*;TH-IRES-Cre*) [43]. However, only recently evidence from studies involving recombinant p62 in vitro [55, 56] and recombinant p62 expressed by an adeno-associated virus vector injected into the rat substantia nigra [57] have suggested that p62 itself is sufficient to form filaments. The structure and the dimensions of the p62 filaments in neurons and neuroepithelial cells of *Atg7*^*f/f*^ *Tyr-Cre* mice is similar or identical to those reported for pure p62 [55] indicating that endogenous p62 is the main, if not the only, component of these filaments. Our double-immunofluorescence labeling results suggest that ubiquitin is also present at elevated concentrations in p62 bodies of *Atg7*^*f/f*^ *Tyr-Cre* mice; however, the relative abundance inside versus outside of these aggregates was much lower for ubiquitin than p62. Furthermore, besides ubiquitin-positive aggregates, apparently ubiquitin-negative aggregates were detected, suggesting that ubiquitinated proteins do not play an essential role in p62 body formation. Thus, we propose that the formation of p62 bodies in *Atg7*^*f/f*^ *Tyr-Cre* mice is likely driven by an inherent tendency to filamentous polymerization of p62 at elevated concentrations.

Our demonstration of elevated amounts of the oxidized phospholipids PAzPC and SAzPC in *Atg7*^*f/f*^ *Tyr-Cre* brains indicates that the turnover of these substances is altered. The differential effects of autophagy inhibition on different classes of oxidized phospholipids (PAzPC and SAzPC versus Lyso-PPC, Lyso-SPC, PLPC, and SLPC in the *Atg7*^*f/f*^ *Tyr-Cre* brain), which are similar to those observed in *Atg7*^*f/f*^ *K14-Cre* skin cells [58], may be caused by the delivery of substrates to lysosomal phospholipase A2 [59]. Augmentation of oxidatively modified phospholipids in cellular membranes affects their polarity and permeability, which has been specifically demonstrated for PLPC-derived PazPC [60]. Additionally, PAzPC has been identified as a chemical modifier of proteins in oxidative stress [32], and as inducer of amyloid fibril aggregation [61]. Further research will address possible mechanistic links between phospholipid oxidation and the protein aggregates in the brain.

In summary, our results suggest that *Tyr-Cre*-mediated suppression of *Atg7*-dependent autophagy causes the aggregation of p62 in neurons and neuroepithelial cells and an increase of oxidized lipid species in the brain. Because of the apparently slow accumulation of damage, *Atg7*^*f/f*^ *Tyr-Cre* mice can be used to study the effects of p62 aggregation in neuroectoderm-derived cells during aging.

## Electronic supplementary material

ESM 1 (PDF 394 kb).

ESM 2 (PDF 2251 kb).

ESM 3 (PDF 521 kb).

ESM 4 (PDF 264 kb).

ESM 5 (PDF 356 kb).

ESM 6 (GIF 774 kb).

High Resolution Image (TIFF 3711 kb).

ESM 7 (GIF 781 kb).

High Resolution Image (TIFF 4193 kb).
