# Supplementary material for: Filamentous Aggregation of Sequestosome-1/p62 in Brain Neurons and Neuroepithelial Cells upon Tyr-Cre-Mediated Deletion of the Autophagy Gene Atg7
Source: Mol Neurobiol. 2018 Mar 17;55(11):8425–37. doi: 10.1007/s12035-018-0996-x (PMC6153718; doi:10.1007/s12035-018-0996-x)
Supplement: Supplementary file 1 — (PDF 394 kb). [file 12035_2018_996_MOESM1_ESM.pdf]

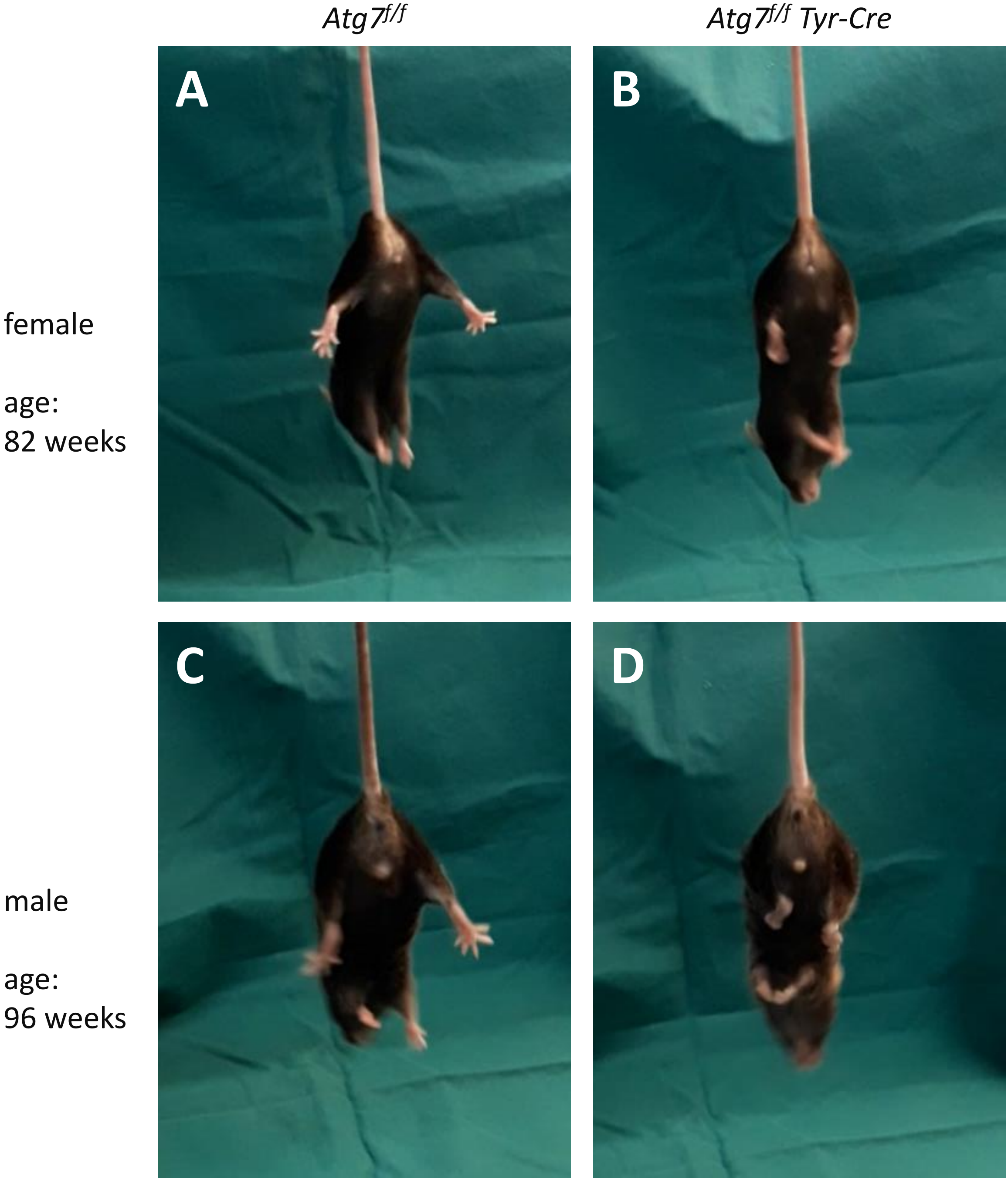

Supplementary Figure S1. Abnormal hindlimb clasping reflexes in *Atg7*$^{f/f}$ *Tyr-Cre* mice. *Atg7*$^{f/f}$ (**A**, **C**) and *Atg7*$^{f/f}$ *Tyr-Cre* (**B**, **D**) mice older than 1.5 years were suspended by their tails. *Atg7*$^{f/f}$ (**A**, **C**) mice extended their hindlimbs which is the normal reflex. *Atg7*$^{f/f}$ *Tyr-Cre* (**B**, **D**) mice showed hindlimb clasping reflexes equivalent to those reported for mouse models of neurodegeneration (Mangiarini et al., 1996). None of 7 *Atg7*$^{f/f}$ and 5 out of 6 *Atg7*$^{f/f}$ *Tyr-Cre* mice showed this abnormal reflex. In contrast to mice lacking *Atg5* or *Atg7* in all neurons (Hara et al., 2006; Komatsu et al., 2006), *Atg7*$^{f/f}$ *Tyr-Cre* mice did not show this defect at young age and at an age of 8 months (n=4).
