# Supplementary material for: Filamentous Aggregation of Sequestosome-1/p62 in Brain Neurons and Neuroepithelial Cells upon Tyr-Cre-Mediated Deletion of the Autophagy Gene Atg7
Source: Mol Neurobiol. 2018 Mar 17;55(11):8425–37. doi: 10.1007/s12035-018-0996-x (PMC6153718; doi:10.1007/s12035-018-0996-x)
Supplement: Supplementary file 2 — (PDF 2251 kb). [file 12035_2018_996_MOESM2_ESM.pdf]

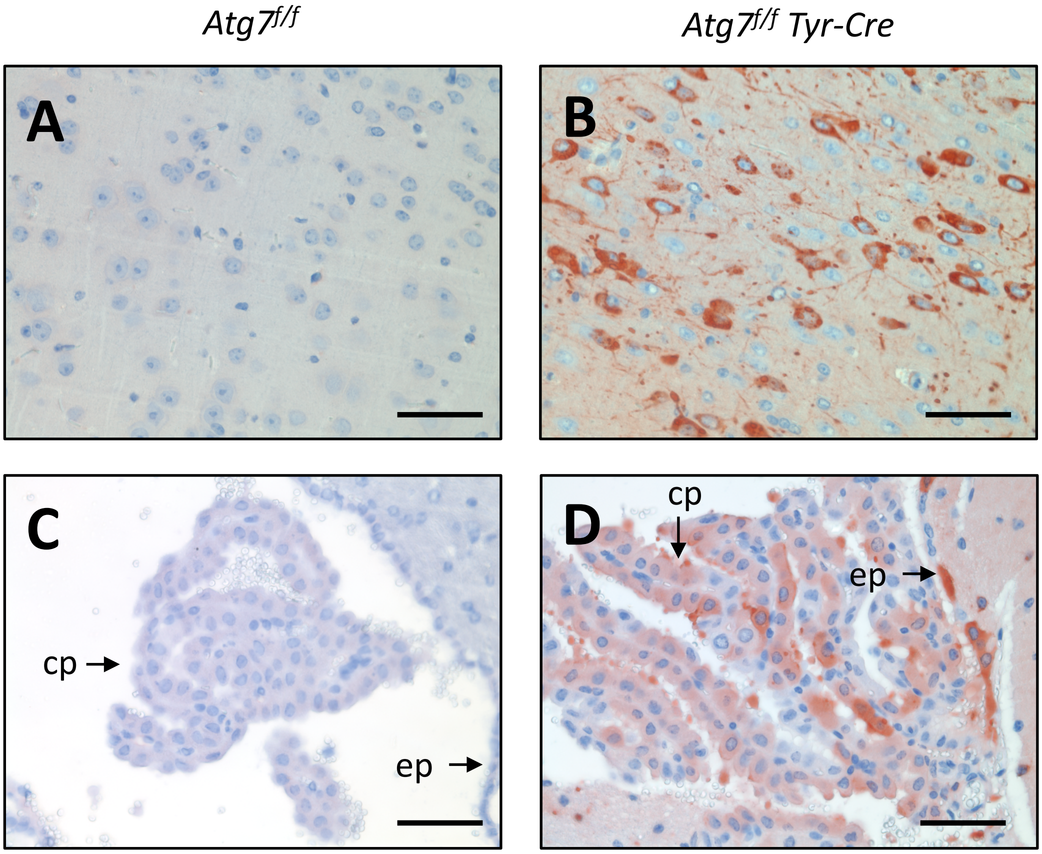

Supplementary Figure S2. p62 accumulation begins within the first month of life in *Atg7$^{f/f}$ Tyr-Cre* mice. p62 was immunohistochemically stained (red) in the brains of 1 month-old *Atg7$^{f/f}$* (**A**, **C**) and *Atg7$^{f/f}$ Tyr-Cre* (**B**, **D**) mice. cp, choroid plexus; ep, ependyma. Scale bars, 50 µm.
