# Supplementary material for: Filamentous Aggregation of Sequestosome-1/p62 in Brain Neurons and Neuroepithelial Cells upon Tyr-Cre-Mediated Deletion of the Autophagy Gene Atg7
Source: Mol Neurobiol. 2018 Mar 17;55(11):8425–37. doi: 10.1007/s12035-018-0996-x (PMC6153718; doi:10.1007/s12035-018-0996-x)
Supplement: Supplementary file 3 — (PDF 521 kb). [file 12035_2018_996_MOESM3_ESM.pdf]

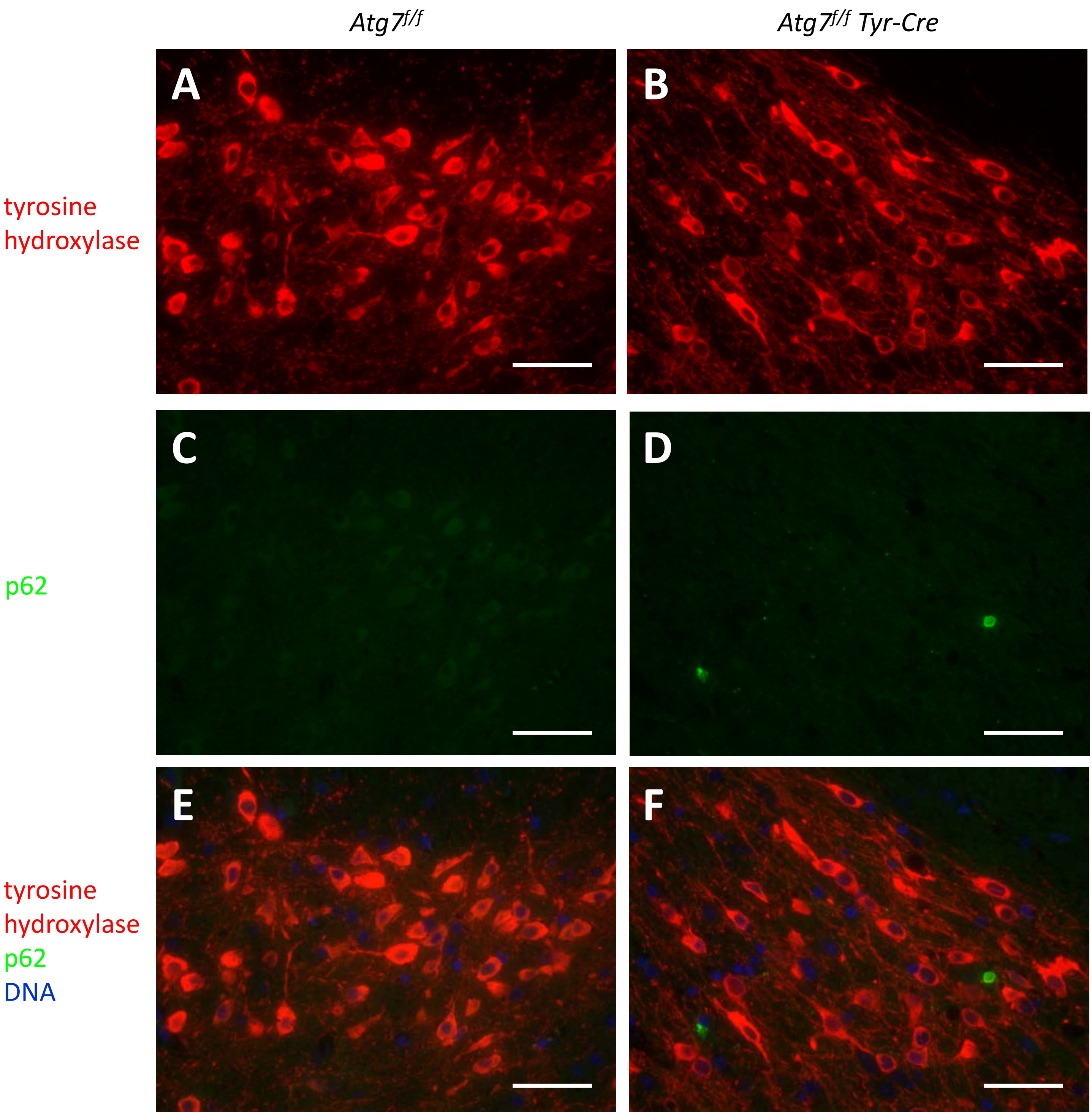

Supplementary Figure S3. p62 does not accumulate in tyrosinase hydroxylase-positive cells of the substantia nigra. Brains of *Atg7*$^{f/f}$ (**A**, **C**, **E**) and *Atg7*$^{f/f}$ *Tyr-Cre* (**B**, **D**, **F**) mice were sectioned and subjected to double immunolabeling for tyrosine hydroxylase (red), a marker of dopaminergic neurons in the substantia nigra, and p62 (green). Nuclear DNA was labeled with Hoechst 33258 dye (blue). Scale bars, 50 µm.
