# Supplementary material for: Filamentous Aggregation of Sequestosome-1/p62 in Brain Neurons and Neuroepithelial Cells upon Tyr-Cre-Mediated Deletion of the Autophagy Gene Atg7
Source: Mol Neurobiol. 2018 Mar 17;55(11):8425–37. doi: 10.1007/s12035-018-0996-x (PMC6153718; doi:10.1007/s12035-018-0996-x)
Supplement: Supplementary file 4 — (PDF 264 kb). [file 12035_2018_996_MOESM4_ESM.pdf]

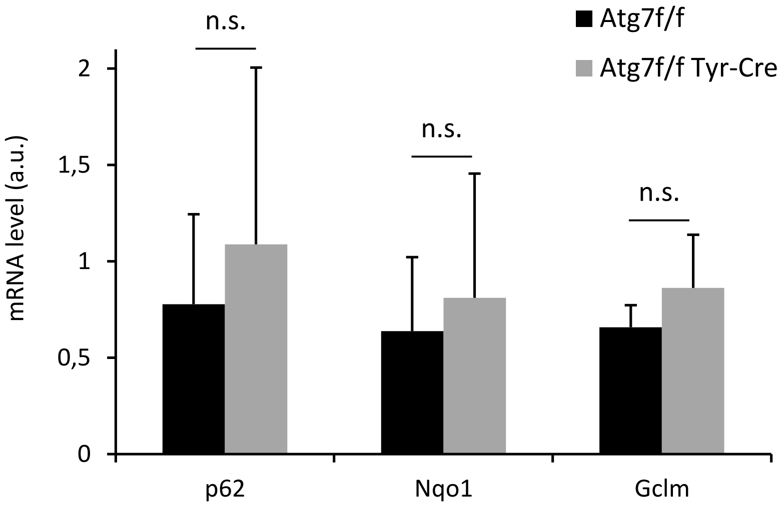

Supplementary Figure S4. Tyr-Cre-mediated suppression of autophagy does not induce transcriptional upregulation of p62 or two other Nrf2 target genes. Reverse transcription quantitative polymerase chain reaction (RT-qPCR) analysis of *Sqstm1/p62*, *Nqo1*, and *Gclm* in brains of *Atg7$^{f/f}$ Tyr-Cre* and *Atg7$^{f/f}$* mice (n=4 per genotype). The mRNA levels were normalized to the abundance of mRNA of the housekeeping gene *B2m*. a.u., arbitrary units; n.s., not significant (2-sided t-test).
