# Supplementary material for: Filamentous Aggregation of Sequestosome-1/p62 in Brain Neurons and Neuroepithelial Cells upon Tyr-Cre-Mediated Deletion of the Autophagy Gene Atg7
Source: Mol Neurobiol. 2018 Mar 17;55(11):8425–37. doi: 10.1007/s12035-018-0996-x (PMC6153718; doi:10.1007/s12035-018-0996-x)
Supplement: Supplementary file 5 — (PDF 356 kb). [file 12035_2018_996_MOESM5_ESM.pdf]

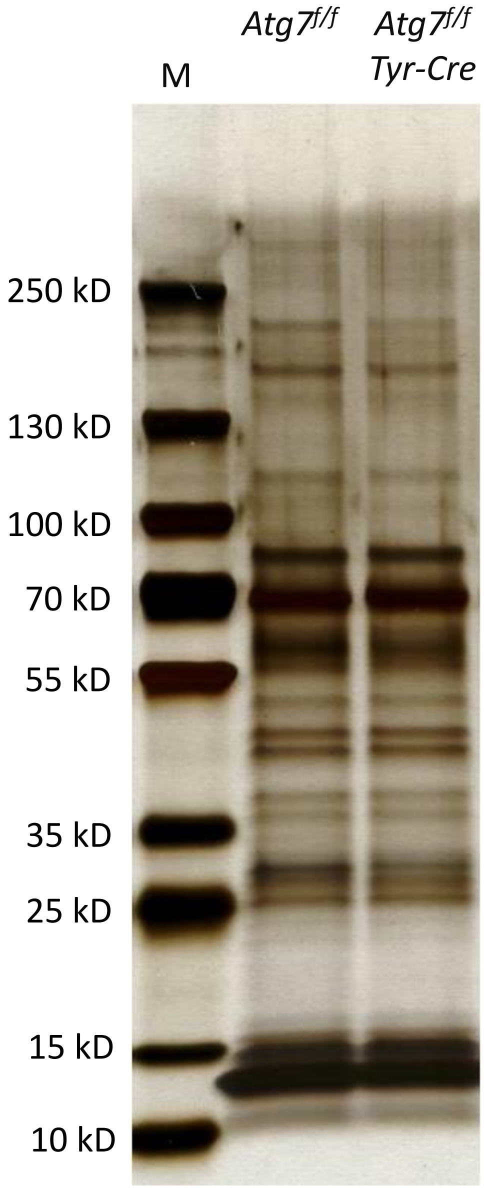

Supplementary Figure S5. Protein composition of the cerebrospinal fluid (CSF) of *Atg7*$^{f/f}$ and *Atg7*$^{f/f}$ *Tyr-Cre* mice. CSF was prepared from *Atg7*$^{f/f}$ and *Atg7*$^{f/f}$ *Tyr-Cre* mice. Proteins were separated by SDS-polyacrylamide electrophoresis and detected by silver-staining. Results are representative for n=3 mice per genotype. The positions of molecular weight markers (M) are indicated on the left. kD, kilo-Dalton.
