# Supplementary figures and images for: Filamentous Aggregation of Sequestosome-1/p62 in Brain Neurons and Neuroepithelial Cells upon Tyr-Cre-Mediated Deletion of the Autophagy Gene Atg7
Source: Mol Neurobiol. 2018 Mar 17;55(11):8425–37. doi: 10.1007/s12035-018-0996-x (PMC6153718; doi:10.1007/s12035-018-0996-x)

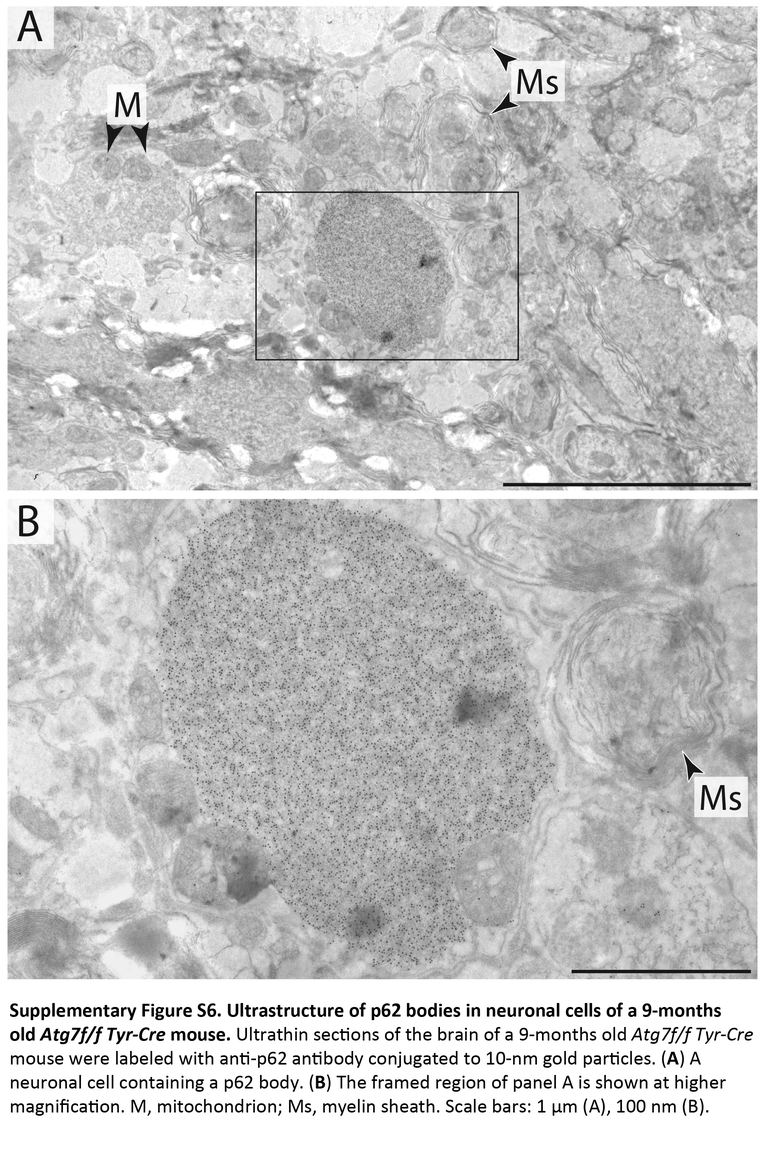

Supplement: Supplementary file 6 — (GIF 774 kb). [file 12035_2018_996_Fig8_ESM.gif]

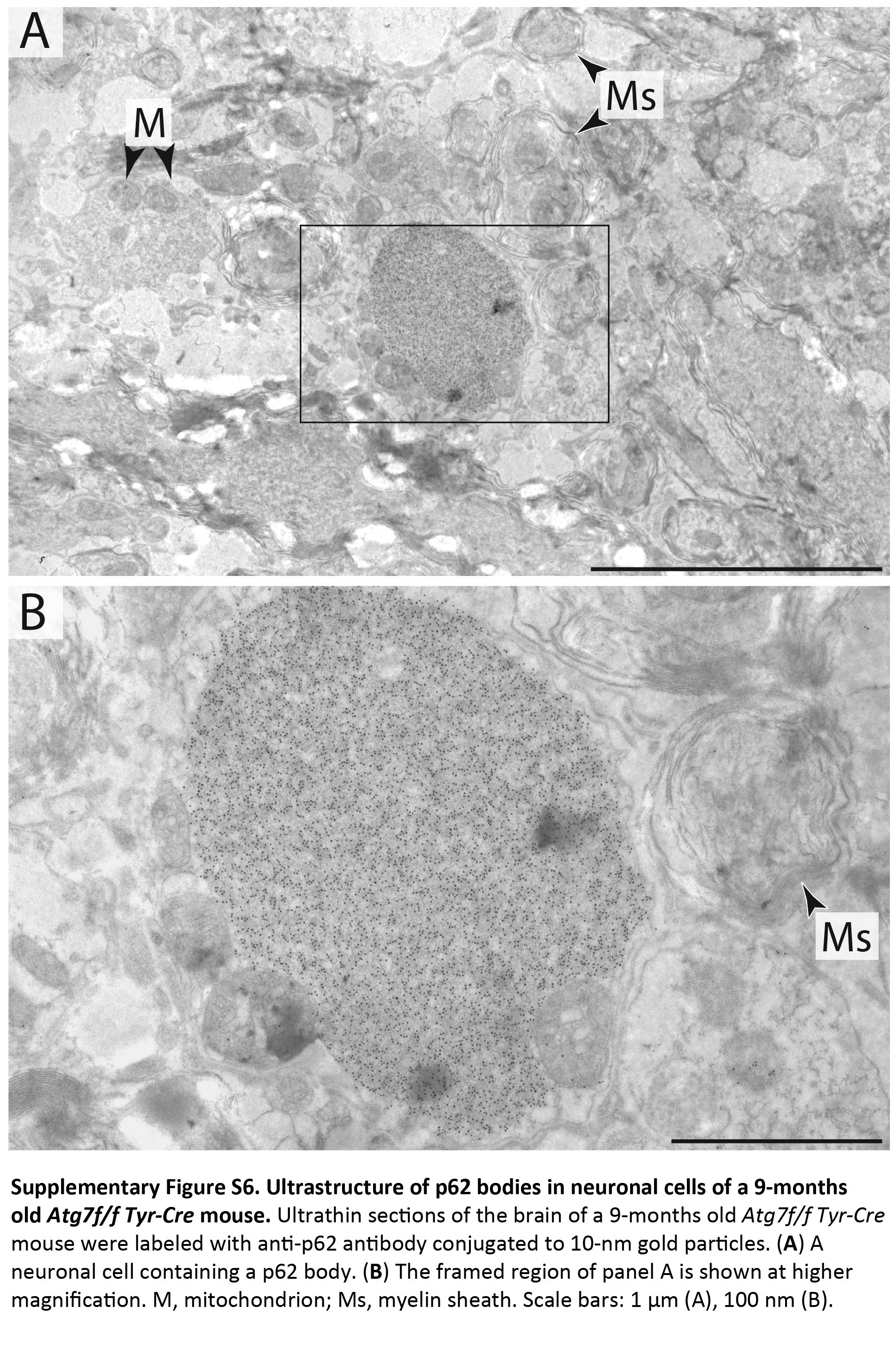

Supplement: Supplementary file 7 — High Resolution Image (TIFF 3711 kb). [file 12035_2018_996_MOESM6_ESM.tif]

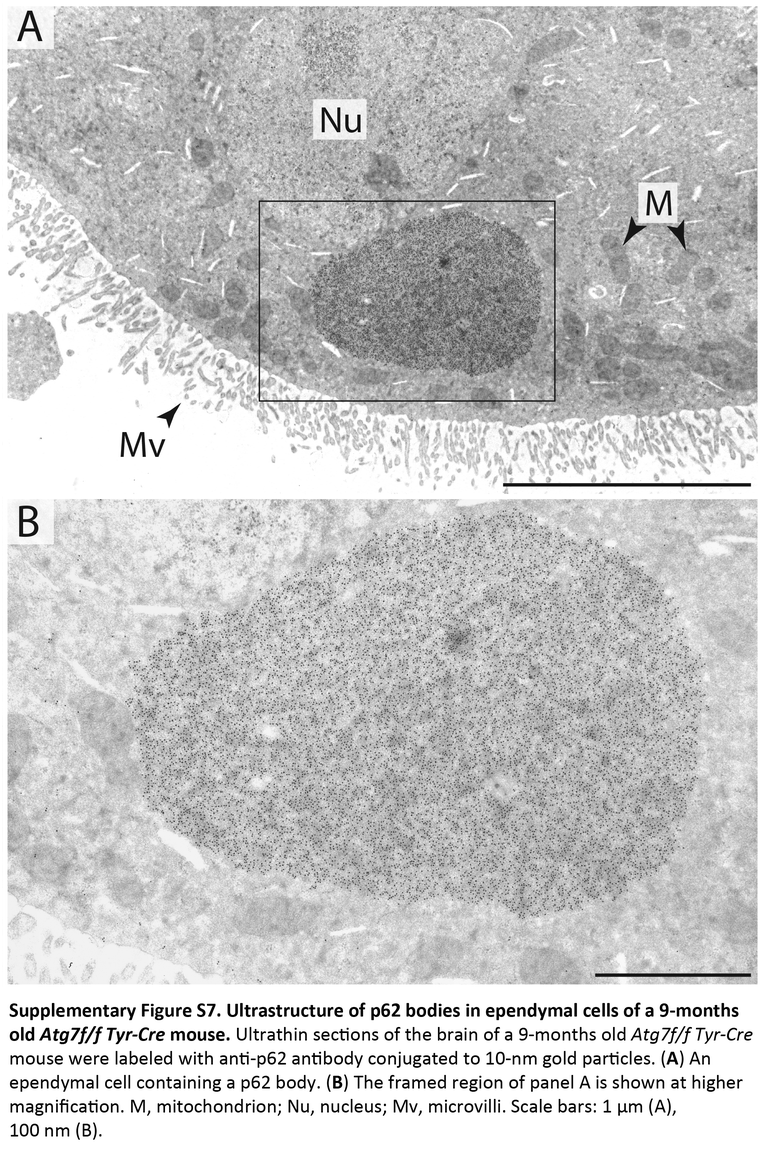

Supplement: Supplementary file 8 — (GIF 781 kb). [file 12035_2018_996_Fig9_ESM.gif]

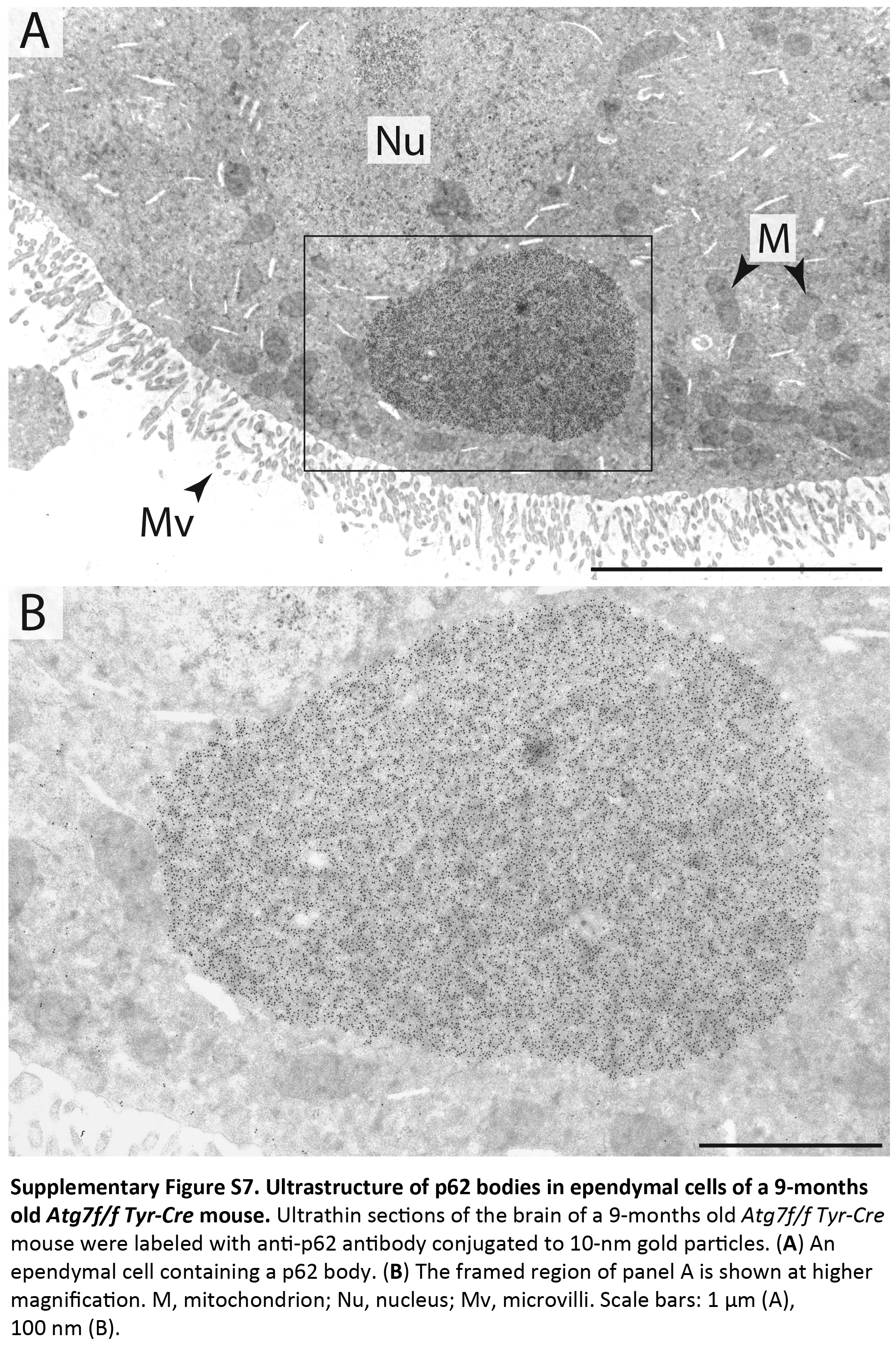

Supplement: Supplementary file 9 — High Resolution Image (TIFF 4193 kb). [file 12035_2018_996_MOESM7_ESM.tif]
